# A longitudinal study of incident hypertension and its determinants in Indian adults aged 45 years and older: evidence from nationally representative WHO-SAGE study (2007–2015)

**DOI:** 10.3389/fcvm.2023.1265371

**Published:** 2023-11-14

**Authors:** Mrigesh Bhatia, Priyanka Dixit, Manish Kumar, Laxmi Kant Dwivedi

**Affiliations:** ^1^ Department of Health Policy, London School of Economics, London, United Kingdom; ^2^ Centre for Health and Social Sociences, Tata Institute of Social Sciences, Mumbai, India; ^3^ Population Research Centre, Dharwad, India; ^4^ Department of Survey Research and Data Analytics, International Institute for Population Sciences, Mumbai, India

**Keywords:** incident hypertension, longitudinal study, pre-hypertension, adults aged 45 years and older, WHO-SAGE, India

## Abstract

**Objectives:**

Hypertension (HT) is a leading cause of mortality and morbidity in developing countries. This study aimed to estimate the incidence of HT among adults aged 45 years and older in India and its associated risk factors.

**Methods:**

This study used longitudinal data from the Indian sample of the first and second waves of the World Health Organization Study on Global Ageing and Adult Health (WHO-SAGE). A bivariate analysis using Pearson's chi-square test was done to examine the associations of individual, lifestyle, and household characteristics with HT status reported in Wave 2. Incident HT changes were analyzed by adjusting for various covariates in the generalized estimating equation (logit link function) with an exchangeable correlation matrix and robust standard errors.

**Results:**

The study found that during the 8-year period from 2007 to 2015, the incidence of HT in individuals aged 45 years and over was 20.8%. Pre-hypertensive individuals had an overall incidence rate of 31.1 per 1,000 [95% confidence interval (CI): 26.20–35.9] and a 2.24 times higher odds ratio: 2.24 (95% CI: 1.65–3.03) of developing incident HT compared to those who were normotensive. Adults aged 45 years and older, overweight/obese individuals, and women were more at risk of incident HT.

**Conclusion:**

One in five individuals had developed HT over 8 years, with a greater risk of incident HT among women than men. Pre-hypertensive individuals were at a greater risk of developing incident HT compared to normotensive individuals. The study recommends comprehensive and effective management of pre-HT to tackle the burden of HT.

## Background

The WHO reported that cardiovascular diseases (CVDs) are the leading cause of death globally, a majority of which occur in low- and middle-income countries (LMICs) (1). Although the prevalence of hypertension (HT) and deaths due to CVDs is on the decline in the developed world, the prevalence is on the rise in LMICs (2). HT is a major risk factor and a leading contributor to premature mortality and morbidity due to CVDs, cerebrovascular events, retinopathy, and chronic renal diseases (3, 4). Globally, an estimated 10.7 million all-cause fatalities in 2015 were related to systolic BP ≥110–115 mmHg, and 7.8 million deaths were related to systolic BP ≥140 mmHg (5).

India—the most populous country with a growing economy (6), an aging population, and increasing urbanization—is also witnessing an increasing prevalence of HT over the years (7) similar to any other LMIC. The United Nations Population Fund projects that the elderly population in India is set to nearly double, reaching a staggering 192 million by the year 2030 (8). A meta-analysis of research conducted between 2001 and 2020 revealed that 24.2% of the Indian population had HT, with 46.8% of those individuals being aware of their elevated blood pressure (9). HT plays a significant role in premature morbidity and mortality in India and contributes to 57% and 24% of all deaths due to stroke and coronary heart disease (CHD) in India (10). Due to demographic shifts, epidemiological changes, and shifts in lifestyle, the prevalence of HT and CVDs in India is projected to increase substantially and is expected to place even greater strain on the healthcare systems of the country that are already under-resourced. The hypertensive Indian population is projected to increase from 20.9% in 2000 to 23.6% in 2025 among women and from 20.6% to 22.9% among men (11).

Although routinely collected clinical data are used for medical research and provide benefits in terms of time, effort, and costs, such data may not be very reliable, especially in the case of India, as it is largely dependent on access to health services and the ability of the people to pay. Moreover, there are several challenges in the use of routine data for specific research purposes.

Several studies have reported the increasing prevalence rates of HT. Although prevalence data have their own merits, they have limitations with respect to chronic conditions such as HT as it is influenced by the duration of the disease. Chronic conditions can persist for years or even decades, and prevalence figures may not reflect changes in disease management, mortality, or treatment effectiveness over time. However, both incidence and prevalence data are useful in policy-making as they help assess the risk and the burden of HT and have their own strengths and limitations.

Given the aging Indian population and huge inequalities in access to health services, which are largely characterized by out-of-pocket expenditure, reliance on routine/prevalence data is likely to provide biased estimates. What would be beneficial to policymakers for planning and implementing preventive strategies is estimating the incidence of new cases of HT. Incident data on HT are currently lacking in many LMICs, including India. In addition, given the increasing burden of HT and CVDs in India, it is necessary to identify the risk factors associated with incident HT. Studies have shown that several risk factors—including aging; rapid urbanization; pre-HT; unhealthy diet; lifestyle changes such as low physical activity, smoking, and alcohol consumption; and an increasing prevalence of obesity and diabetes—are likely to contribute to a rapid rise in HT (12).

Longitudinal studies help identify the development of new cases of HT over time and are more effective in targeting resources for those at risk and implementing preventive strategies at the primary level. A prospective study, namely, the World Health Organization Study on Global Ageing and Adult Health (WHO-SAGE) conducted in two waves (2007 and 2015), provides an excellent opportunity to explore the risk factors associated with the incidence of HT. To the best of our knowledge, there are hardly any studies that have reported incident data on HT and its determinants based on a longitudinal nationally representative data set in the context of LMICs, and India is no exception to this.

Therefore, this study aims to quantify the incidence of HT in adults aged 45 years and older in India and identify the risk factors associated with incident HT. This study not only contributes to the empirical knowledge on incident HT from an LMIC perspective but also has significant implications for health policy in terms of implementing effective preventive strategies at the primary level.

## Methods

### Study design and participants

The present study used longitudinal data from the Indian sample of the first and second waves of the WHO-SAGE. SAGE is an ongoing, nationally representative longitudinal survey conducted in six LMICs, namely, China, Ghana, India, Mexico, South Africa, and the Russian Federation. Our study focused on individuals who were aged 45 years and older in Wave 2, with baseline individuals aged 37 years and older (due to the 8-year gap between the two waves). The decision to prioritize individuals aged 45 years and older was made with the explicit aim of targeting the aging population as this age cohort is more prone to health issues, including HT. The sample for SAGE India Wave 2 (2015) was the follow-up survey of Wave 1 (2007) in India. The primary purpose of the SAGE India survey was to collect information related to the health and wellbeing of the growing aging population of India.

The survey design of WHO-SAGE incorporated specific primary sampling units (PSUs) and households from the 2003 World Health Survey in India to establish a baseline sample. The survey design covered six Indian states, namely, Karnataka, Assam, Maharashtra, Uttar Pradesh, Rajasthan, and West Bengal. The selection of these states was determined by a combination of factors such as geographical diversity and development indicators such as infant mortality rate, literacy rate of women, percentage of safe deliveries, and per capita income. These states were deliberately chosen to represent various regions and levels of development.

The sampling approaches differed for rural and urban areas. Rural sampling employed a two-stage stratified method, categorizing villages based on household numbers and then employing probability proportional to size sampling for village selection. Each PSU yielded a selection of 25 households. In urban areas, a three-stage approach was used, involving the selection of wards, census enumeration blocks, and households. Cities/towns were grouped into four categories based on the 1991 census population for this purpose (13).

The initial merged sample of Wave 1 and Wave 2 consisted of 3,630 individuals. However, individuals with missing information on any of the variables of interest were excluded from the final data (*n* = 447). Therefore, our final sample included 3,183 individuals followed in Waves 1 and 2.

### Procedures

The blood pressure was measured in both the waves in the right arm/wrist using an OMRON R6 wrist blood pressure monitor using a standard protocol by trained health workers. In this study, the average of the last two readings of the systolic blood pressure (SBP) and the diastolic blood pressure (DBP) was considered for the analysis (14, 15). According to the JNC-7 guidelines, a person was considered hypertensive if he/she had an average SBP ≥140 mmHg or/and an average DBP ≥90 mmHg or was a current user of anti-hypertensive medication at the time of the survey (16). An individual was identified as pre-hypertensive if his/her SBP was 120–139 mmHg and/or if his/her DBP was 80–89  mmHg (16). The 8-year incident cases of HT were defined as those who were free of HT in the first wave but had developed HT by the time the follow-up was conducted. Healthcare utilization was defined as a visit to a healthcare facility as an inpatient or an outpatient at least once during the 12 months prior to the survey.

A set of individual, lifestyle, and household characteristics were included in the analysis to assess the determinants of the incident cases of HT. The individual-level factors were sex (men, women), age group (<45, 45–54, 55–64, 65–74, 75+ years), working status (never worked, not currently working, currently working), marital status (currently married, others), body mass index (BMI) [normal (18.5–24.9 kg/m²), underweight (<18.5 kg/m²), overweight/obese (≥25.0 kg/m^2^)] (17), stroke (no, yes), diabetes (no, yes), and chronic lung disease (no, yes).

In this study, we grouped the education levels of individuals into four categories, i.e., “no education,” “primary,” “secondary,” and “higher.” These groupings were determined based on the highest formal education level of the individual, as assessed through two questions from the WHO-SAGE India questionnaire. The initial question asked, “Have you ever attended school?” with two possible responses: (1) “yes” and (2) “no.” If an individual had never attended school, they were categorized as having “no education.” Subsequently, a follow-up question inquired, “What is the highest level of education that you have completed?” This question had six possible responses: (1) “less than primary school,” (2) “primary school completed,” (3) “secondary school completed,” (4) “high school (or equivalent) completed,” (5) “college/pre-university/university completed,” and (6) “postgraduate degree completed.” To simplify our analysis, we merged categories (1) “less than primary school” and (2) “primary school completed” into a single category called “primary.” Furthermore, we reclassified category (3) “high school (or equivalent) completed” as “secondary.” Finally, we combined the categories (4) “college/pre-university/university completed” and (5) “postgraduate degree completed” into the “higher” category.

Moreover, the WHO-SAGE Wave-2 questionnaire includes a section dedicated to assessing physical activity, specifically aimed at gathering information regarding activities of moderate and vigorous intensity. Two questions were posed to the respondents, one inquiring about their participation in vigorous-intensity activities that result in significant increases in breathing or heart rate, such as heavy lifting, digging, or chopping wood, and the other inquiring about their engagement in moderate-intensity activities that lead to slight increases in breathing or heart rate, such as brisk walking, carrying light loads, cleaning, cooking, or washing clothes. These questions are designed with binary response options, requiring participants to answer either “yes” or “no.” To classify physical activity, we adopted the criteria proposed by Peltzer and Phaswana-Mafuya (18). Other lifestyle factors included tobacco use (never, former, occasional, current) and alcohol use (no, yes). It may be noted that the prevalence of stroke, diabetes, and chronic lung disease are self-reported.

Wealth quintiles were determined based on household ownership of durable goods and dwelling characteristics. For example, durable goods include factors such as the number of motorbikes or cars and the presence of amenities such as electricity, a television, fixed-line or mobile phone, and a bucket or washing machine. The analysis used for determining the wealth index can be found in the WHO-SAGE India Wave 2 report (13). The household-level characteristics included wealth quintiles (poorest, poorer, middle, richer, richest), caste [scheduled caste (SC)/scheduled tribe (ST), other backward class (OBC), others], religion (Hinduism, Islam, others), and place of residence (urban, rural).

The “caste” reported by an interviewed individual typically falls into one of four main categories: SC, ST, OBC, and “general class.” In India, SC and ST communities are widely recognized as some of the most socioeconomically disadvantaged groups. Conversely, individuals belonging to the general class occupy a higher hierarchical social status. The OBCs, while facing educational, economic, and social challenges, hold a relatively better social standing when compared to the SC and ST categories from a hierarchical perspective.

### Statistical analysis

To estimate the incidence rate of HT, new cases that emerged between Waves 1 and 2 of the WHO-SAGE were identified. All non-hypertensives at baseline who were found to have HT in Wave 2 were assumed to have developed HT at the time of follow-up. The incidence rate was then calculated by dividing the number of incident cases by the total number of individuals at the baseline (WHO-SAGE Wave 1) and was expressed per 1,000 people. We provided confidence intervals to address estimation uncertainty. The sample characteristics were expressed as the percentages of the categorical explanatory variables for both men and women participants. The chi-square test was used to compare the distribution of the categories between men and women. Bivariate analysis using Pearson's chi-square test was used to examine the associations of individual, lifestyle, and household characteristics with HT status in Wave 2. Categorical variable (incident HT) changes were analyzed by incorporating various covariates in a binomial logistic regression model. Odds ratios (ORs), 95% confidence intervals (CIs), and *p*-values were estimated using the generalized estimating equation (GEE) (logit link function) with an exchangeable working correlation matrix and robust standard errors (19). The prevalence of inpatient and outpatient healthcare utilization by HT status was also estimated. The Stata software (version 16.1) was used to execute all the data analysis. A *p*-value of less than 0.05 was considered statistically significant for all the analyses.

### Role of the funding source

The funders of the study had no role in the study design, data collection, data analysis, data interpretation, or the writing of the manuscript.

## Results

The differences in the sociodemographic characteristics of the included and excluded samples in the study are presented in Supplementary Table S1 (Supplementary Material). The findings indicate that there were no significant disparities between the excluded and included samples in terms of various background attributes, including age group, gender, education, and employment status. Table 1 shows the baseline characteristics and the gender of the overall sample. A total of 3,183 individuals were included in the analysis, of whom 1,513 were men and 1,670 were women. More women than men had no education (65.2% vs. 27.8%), had never worked (47.5% vs. 2.2%), were either unmarried or widowed (29.5% vs. 7.9%), and were overweight/obese (17.7% vs. 10.6%). In contrast, the prevalence of various chronic conditions such as stroke (1.7% vs. 1.3%), diabetes (5.9% vs. 3.4%), and chronic lung disease (4.2% vs. 2.0%) was greater among men than women. Going by lifestyle factors, the prevalence of vigorous activity (37.0% vs. 14.7%), current tobacco use (64.6% vs. 26.6%), and alcohol use (29.9% vs. 3.2%) was higher in men than in women. Nearly 84.2% of the individuals belonged to the Hindu religion, and approximately 79.5% of all individuals were rural residents.

**Table 1:** Sample characteristics, WHO-SAGE baseline, India.

Baseline background characteristics	Overall (*n* = 3,183)		Men (*n* = 1,513)		Women (*n* = 1,670)		*p*-value
	*N*	%	*N*	%	*N*	%	
Individual factors							
Age groups (years)							
<45	523	16.4	209	13.8	314	18.8	*p* < 0.001
45–54	994	31.2	430	28.4	564	33.8	
55–64	1,083	34.0	546	36.1	537	32.2	
65–74	508	16.0	279	18.4	229	13.7	
75+	75	2.4	49	3.2	26	1.6	
Education							
No education	1,510	47.4	421	27.8	1,089	65.2	*p* < 0.001
Primary	854	26.8	485	32.1	369	22.1	
Secondary	356	11.2	250	16.5	106	6.3	
Higher	463	14.5	357	23.6	106	6.3	
Working status							
Never worked	827	26.0	33	2.2	794	47.5	*p* < 0.001
Not currently working	705	22.1	336	22.2	369	22.1	
Currently working	1,651	51.9	1,144	75.6	507	30.4	
Marital status							
Currently married	2,570	80.7	1,393	92.1	1,177	70.5	*p* < 0.001
Others	613	19.3	120	7.9	493	29.5	
BMI categories (kg/m^2^)							
Normal (18.5–24.9)	1,680	52.8	855	56.5	825	49.4	*p* < 0.001
Underweight (≤18.4)	1,046	32.9	497	32.8	549	32.9	
Overweight/obese (≥25)	457	14.4	161	10.6	296	17.7	
Stroke							
No	3,136	98.5	1,487	98.3	1,649	98.7	*p* < 0.282
Yes	47	1.5	26	1.7	21	1.3	
Diabetes							
No	3,037	95.4	1,424	94.1	1,613	96.6	*p* = 0.001
Yes	146	4.6	89	5.9	57	3.4	
Chronic lung disease							
No	3,086	97.0	1,449	95.8	1,637	98.0	*p* < 0.001
Yes	97	3.0	64	4.2	33	2.0	
Lifestyle factors							
Moderate activity							
No	2,088	65.6	1,037	68.5	1,051	62.9	*p* = 0.001
Yes	1,095	34.4	476	31.5	619	37.1	
Vigorous activity							
No	2,377	74.7	953	63.0	1,424	85.3	*p* < 0.001
Yes	806	25.3	560	37.0	246	14.7	
Tobacco use							
Never	1,564	49.1	374	24.7	1,190	71.3	*p* < 0.001
Former	116	3.6	101	6.7	15	0.9	
Occasional	80	2.5	60	4.0	20	1.2	
Current	1,423	44.7	978	64.6	445	26.6	
Alcohol use							
No	2,676	84.1	1,060	70.1	1,616	96.8	*p* < 0.001
Yes	507	15.9	453	29.9	54	3.2	
Household factors							
Wealth quintiles							
Poorest	723	22.7	333	22.0	390	23.4	*p* = 0.542
Poorer	711	22.3	337	22.3	374	22.4	
Middle	617	19.4	283	18.7	334	20.0	
Richer	608	19.1	303	20.0	305	18.3	
Richest	524	16.5	257	17.0	267	16.0	
Caste							
SC/ST	811	25.5	382	25.2	429	25.7	*p* = 0.385
OBC	1,887	59.3	913	60.3	974	58.3	
Others	485	15.2	218	14.4	267	16.0	
Religion							
Hinduism	2,680	84.2	1,248	82.5	1,432	85.7	*p* = 0.029
Islam	378	11.9	195	12.9	183	11.0	
Others	125	3.9	70	4.6	55	3.3	
Place of residence							
Urban	652	20.5	271	17.9	381	22.8	*p* = 0.001
Rural	2,531	79.5	1,242	82.1	1,289	77.2	
Total	3,183	100.0	1,513	100.0	1,670	100.0	

SC/ST, schedule caste/schedule tribe; OBC, other backward classes; BMI, body mass index.

*p*-values have been computed using the chi-square test to assess the distributional differences between men and women across categories.

The bivariate analysis of various individual, lifestyle, and household characteristics with the HT status (normal and pre-hypertensive) at the baseline is presented in Table 2. The weighted prevalence of pre-HT at the baseline was 38.2% (unweighted *N* = 1,086). A greater proportion of pre-hypertensive individuals than normotensive individuals were older, less educated, currently working, and current tobacco users and had a normal body mass index. We did not find any bivariate association of chronic conditions such as stroke, diabetes, and chronic lung disease with HT status.

**Table 2:** Bivariate analysis of hypertension status with various individual, lifestyle, and household characteristics at baseline, WHO-SAGE, India.

Baseline background characteristics	Overall (*n* = 2,430)		Normal (*n* = 1,344)		Pre-HT (*n* = 1,086)	
	*N*	%	*N*	%	*N*	%
Individual factors						
Age groups (years)	*p = 0.059*					
<45	433	44.1	257	45.7	176	41.4
45–54	767	34.2	442	35.2	325	32.5
55–64	807	15.3	424	13.4	383	18.5
65–74	368	5.5	190	4.7	178	6.8
75+	55	0.9	31	1.0	24	0.7
Sex	*p = 0.227*					
Men	1,172	64.1	663	67.5	509	58.4
Women	1,258	35.9	681	32.5	577	41.6
Education	*p = 0.911*					
No education	1,152	38.1	638	36.3	514	41.2
Primary	650	28.8	362	30.6	288	25.9
Secondary	272	14.2	153	14.4	119	14.0
Higher	356	18.8	191	18.7	165	18.9
Working status	*p = 0.388*					
Never worked	619	17.6	333	16.0	286	20.3
Not currently working	527	12.5	284	12.1	243	13.1
Currently working	1,284	69.9	727	72.0	557	66.6
Marital status	*p = 0.053*					
Currently married	1,966	89.2	1,106	89.8	860	88.3
Others	464	10.8	238	10.2	226	11.7
BMI categories (kg/m^2^)	*p < 0.001*					
Normal (18.5–24.9)	1,265	52.0	656	48.4	609	57.9
Underweight (≤18.4)	850	36.8	543	42.3	307	27.9
Overweight/obese (≥25)	315	11.1	145	9.2	170	14.2
Stroke	*p < 0.299*					
No	2,399	98.9	1,324	99.1	1,075	98.7
Yes	31	1.1	20	0.9	11	1.3
Diabetes	*p < 0.557*					
No	2,325	96.0	1,283	95.6	1,042	96.6
Yes	105	4.0	61	4.4	44	3.4
Chronic lung disease	*p < 0.372*					
No	2,352	96.5	1,297	96.2	1,055	96.9
Yes	78	3.5	47	3.8	31	3.1
Lifestyle factors						
Moderate activity	*p = 0.049*					
No	1,591	63.9	857	61.8	734	67.4
Yes	839	36.1	487	38.2	352	32.6
Vigorous activity	*p = 0.151*					
No	1,798	63.7	979	62.2	819	66.2
Yes	632	36.3	365	37.8	267	33.8
Tobacco use	*p = 0.110*					
Never	1,183	39.7	626	36.1	557	45.5
Former	84	2.6	48	2.4	36	3.0
Occasional	61	2.3	32	1.8	29	3.2
Current	1,102	55.3	638	59.6	464	48.3
Alcohol use	*p = 0.076*					
No	2,060	78.2	1,155	77.4	905	79.4
Yes	370	21.8	189	22.6	181	20.6
Household factors						
Wealth quintiles	*p = 0.010*					
Poorest	571	26.1	338	29.0	233	21.4
Poorer	545	24.2	323	23.7	222	24.9
Middle	465	20.0	246	19.8	219	20.2
Richer	465	17.5	236	16.9	229	18.4
Richest	384	12.3	201	10.6	183	15.1
Caste	*p = 0.068*					
SC/ST	608	23.6	354	25.3	254	20.7
OBC	1,462	63.3	808	62.8	654	64.2
Others	360	13.1	182	11.8	178	15.1
Religion	*p = 0.862*					
Hinduism	2,040	83.5	1,124	83.1	916	84.0
Islam	298	12.3	167	12.2	131	12.3
Others	92	4.3	53	4.7	39	3.7
Place of residence	*p = 0.038*					
Urban	474	20.6	242	20.7	232	20.4
Rural	1,956	79.4	1,102	79.3	854	79.6
Total	2,430	100.0	1,344	100.0	1,086	100.0

SC/ST, schedule caste/schedule tribe; OBC, other backward classes; BMI, body mass index.

*N* is unweighted, and % is weighted.

Table 3 presents the HT status during the follow-up according to the baseline status of HT. During the follow-up period of 8 years, 31.1% of the pre-hypertensive and 14.4% of the normotensive participants had developed HT. Women had a greater level of conversion from normotension and pre-HT status to HT during the follow-up.

**Table 3:** The changes in blood pressure category on follow-up according to baseline blood pressure category, WHO-SAGE, India.

	Blood pressure on follow-up (SAGE Wave 2)		
	Normal	Pre-HT	HT
SAGE Wave 1 (baseline)	*N* (%)	*N* (%)	*N* (%)
Overall (*n* = 2,430)			
Normal (*n* = 1,344)	581 (47.5%)	540 (38.1%)	223 (14.4%)
Pre-HT (*n* = 1,086)	229 (21.2%)	475 (47.7%)	382 (31.1%)
Men (*n* = 1,172)			
Normal (*n* = 663)	273 (46.4%)	288 (40.3%)	102 (13.3%)
Pre-HT (*n* = 509)	104 (19.8%)	239 (50.4%)	166 (29.8%)
Women (*n* = 1,258)			
Normal (*n* = 681)	308 (49.8%)	252 (33.5%)	121 (16.7%)
Pre-HT (*n* = 577)	125 (23.2%)	236 (43.9%)	216 (32.9%)

HT, hypertension.

*N* is unweighted, and % is weighted.

The 8-year incidence rates of HT according to the baseline characteristics are presented in Table 4. The 8-year incidence rate of HT was 20.8%. The rate of HT in normotensive and pre-hypertensive individuals was 14.4% and 31.1%, respectively. The incidence rate of HT in normotensive individuals was greater among individuals who were older, women, educated, unmarried or widowed, overweight/obese, moderately active, vigorously active, and alcohol consumers and who had never used tobacco. The incidence rates hold true for HT in pre-hypertensive participants, except for individuals who did not engage in moderate activity or vigorous activity, had a history of tobacco use, or consumed alcohol.

**Table 4:** Eight-year incidence rates of hypertension (HT) according to baseline blood pressure categories, WHO-SAGE, India.

Baseline background characteristics	Eight-year incidence of HT		
	Normal to hypertensive	Pre-hypertensive to hypertensive	Overall
	%	%	%
Individual factors			
Age groups (years)			
<45	13.5	28.5	18.9
45–54	13.6	30.2	19.7
55–64	16.4	35.0	25.0
65–74	18.1	40.8	28.9
75+	36.0	28.8	33.8
Sex			
Men	13.3	29.8	19.0
Women	16.7	32.9	23.9
Education			
No education	12.6	28.5	19.1
Primary	15.3	28.8	20.0
Secondary	25.3	36.1	29.4
Higher	7.9	36.2	18.7
Working status			
Never worked	17.6	40.2	27.6
Not currently working	18.0	29.1	22.4
Currently working	13.1	28.7	18.8
Marital status			
Currently married	13.7	30.8	20.2
Others	20.4	33.5	25.8
BMI categories (kg/m^2^)			
Normal (18.5–24.9)	15.6	31.1	22.2
Underweight (≤18.4)	9.7	27.2	14.8
Overweight/obese (≥25)	29.2	38.5	33.7
Stroke			
No	14.5	31.4	20.9
Yes	4.1	5.8	4.9
Diabetes			
No	14.3	31.4	20.8
Yes	17.1	22.9	18.9
Chronic lung disease			
No	14.9	31.6	21.3
Yes	1.4	16.9	6.6
Lifestyle factors			
Moderate activity			
No	12.0	31.8	20.0
Yes	18.2	29.6	22.1
Vigorous activity			
No	13.1	33.2	21.1
Yes	16.5	26.9	20.2
Tobacco use			
Never	15.1	29.5	21.4
Former	3.9	80.5	36.8
Occasional	7.1	22.2	15.0
Current	14.6	30.1	19.8
Alcohol use			
No	14.0	30.9	20.5
Yes	15.8	32.0	21.6
Household factors			
Wealth quintiles			
Poorest	9.5	29.7	15.8
Poorer	13.6	21.9	16.9
Middle	19.6	36.7	26.2
Richer	20.5	32.1	25.2
Richest	10.2	39.4	23.8
Caste			
SC/ST	11.8	26.9	16.8
OBC	14.8	29.6	20.6
Others	17.7	42.9	28.8
Religion			
Hinduism	14.2	32.6	21.3
Islam	16.9	20.8	18.4
Others	12.0	30.5	18.1
Place of residence			
Urban	13.5	39.2	23.2
Rural	14.6	29.0	20.1
Total	14.4	31.1	20.8

SC/ST, schedule caste/schedule tribe; OBC, other backward classes; BMI, body mass index.

Table 5 shows the adjusted odds ratio of the incident HT estimated using the GEE (logit link function) according to various individual, lifestyle, and household characteristics. The results suggest that after adjusting for the selected covariates, a greater risk of incidence of HT was observed among the pre-hypertensive participants (OR: 2.24, 95% CI: 1.65–3.03) than the normotensive participants. Similar results were obtained for men and women participants. Wealth status was not significantly associated with the overall incident HT and with incident HT among both men and women participants.

**Table 5:** Adjusted odds ratio obtained from generalized estimating equation (GEE) with an exchangeable correlation matrix of the incidence of hypertension associated with selected covariates, WHO-SAGE, India.

Baseline background characteristics	Overall		Men		Women	
	OR	95% CI	OR	95% CI	OR	95% CI
Individual factors						
Age groups						
<45	*Ref.*		*Ref.*		*Ref.*	
45–54	1.08	(0.74, 1.59)	0.74	(0.42, 1.34)	1.77**	(1.13, 2.78)
55–64	1.27	(0.90, 1.79)	0.91	(0.57, 1.43)	1.84**	(1.11, 3.05)
65–74	1.59**	(1.03, 2.45)	1.01	(0.54, 1.90)	2.30***	(1.25, 4.23)
75+	2.36	(0.84, 6.59)	2.69	(0.62, 11.76)	1.73	(0.47, 6.42)
Baseline BP status						
Normal	*Ref.*		*Ref.*		*Ref.*	
Pre-hypertension	2.24***	(1.65, 3.03)	2.40***	(1.51, 3.81)	2.06***	(1.48, 2.86)
Sex						
Male	*Ref.*					
Female	1.15	(0.72, 1.83)	–	–	–	–
Education						
No education	*Ref.*		*Ref.*		*Ref.*	
Primary	1.15	(0.79, 1.67)	0.95	(0.49, 1.84)	1.64**	(1.12, 2.38)
Secondary	1.80**	(1.12, 2.91)	1.88*	(0.99, 3.55)	0.95	(0.46, 1.96)
Higher	0.99	(0.59, 1.66)	0.9	(0.45, 1.81)	1.14	(0.55, 2.36)
Working status						
Never worked	*Ref.*		*Ref.*		*Ref.*	
Not currently working	0.78	(0.53, 1.16)	2.24	(0.67, 7.47)	0.56***	(0.37, 0.84)
Currently working	0.66**	(0.44, 0.98)	1.14	(0.36, 3.58)	0.73	(0.50, 1.07)
Marital status						
Currently married	*Ref.*		*Ref.*		*Ref.*	
Others	1.2	(0.81, 1.76)	1.37	(0.57, 3.29)	1.08	(0.72, 1.64)
BMI categories						
Normal (18.5–24.9 kg/m^2^)	*Ref.*		*Ref.*		*Ref.*	
Underweight (≤18.4)	0.68**	(0.48, 0.98)	0.68	(0.39, 1.19)	0.78	(0.53, 1.15)
Overweight/obese (≥25)	1.47*	(0.94, 2.28)	1.41	(0.69, 2.88)	1.86***	(1.23, 2.83)
Stroke						
No	*Ref.*		*Ref.*		*Ref.*	
Yes	0.13***	(0.03, 0.57)	0.10**	(0.01, 0.81)	0.21**	(0.05, 0.82)
Diabetes						
No	*Ref.*		*Ref.*		*Ref.*	
Yes	0.84	(0.41, 1.72)	0.67	(0.27, 1.67)	1.66	(0.61, 4.50)
Chronic lung disease						
No	*Ref.*		*Ref.*		*Ref.*	
Yes	0.33**	(0.13, 0.83)	0.06***	(0.01, 0.35)	1.51	(0.63, 3.60)
Lifestyle factors						
Moderate activity						
No	*Ref.*		*Ref.*		*Ref.*	
Yes	1.25	(0.91, 1.71)	1.59*	(0.96, 2.62)	1.09	(0.80, 1.49)
Vigorous activity						
No	*Ref.*		*Ref.*		*Ref.*	
Yes	1.05	(0.73, 1.52)	1.05	(0.64, 1.74)	1.05	(0.69, 1.60)
Tobacco use						
Never	*Ref.*		*Ref.*		*Ref.*	
Former	1.88**	(1.07, 3.28)	1.68	(0.85, 3.32)	1.13	(0.27, 4.76)
Occasional	0.63	(0.22, 1.79)	0.48	(0.13, 1.73)	1.45	(0.26, 8.02)
Current	1.34	(0.93, 1.94)	1.26	(0.71, 2.23)	1.44**	(1.01, 2.04)
Alcohol use						
No	*Ref.*		*Ref.*		*Ref.*	
Yes	1.13	(0.71, 1.78)	1.17	(0.71, 1.94)	0.28**	(0.09, 0.82)
Household factors						
Wealth quintiles						
Poorest	*Ref.*		*Ref.*		*Ref.*	
Poorer	0.91	(0.58, 1.44)	0.97	(0.45, 2.09)	0.76	(0.48, 1.21)
Middle	1.51*	(0.98, 2.34)	1.91*	(0.98, 3.74)	1.15	(0.75, 1.77)
Richer	1.34	(0.87, 2.09)	1.63	(0.82, 3.24)	1.02	(0.62, 1.69)
Richest	1.09	(0.66, 1.82)	1.43	(0.67, 3.04)	0.72	(0.40, 1.29)
Caste						
SC/ST	*Ref.*		*Ref.*		*Ref.*	
OBC	1.11	(0.77, 1.59)	1.41	(0.80, 2.51)	0.75	(0.51, 1.09)
Others	1.63**	(1.02, 2.60)	2.58***	(1.28, 5.20)	0.79	(0.47, 1.33)
Religion						
Hinduism	*Ref.*		*Ref.*		*Ref.*	
Islam	0.83	(0.51, 1.35)	0.73	(0.34, 1.55)	1.02	(0.60, 1.72)
Others	0.99	(0.51, 1.96)	0.84	(0.35, 2.04)	1.08	(0.51, 2.30)
Place of residence						
Urban	*Ref.*		*Ref.*		*Ref.*	
Rural	1.06	(0.73, 1.55)	0.87	(0.49, 1.54)	1.63**	(1.05, 2.52)

OR, odds ratio; CI, confidence interval; Ref., reference group; SC/ST, schedule caste/schedule tribe; OBC, other backward classes.

* *p* ≤ 0.05.

** *p* ≤ 0.01.

*** *p* ≤ 0.001.

## Discussion

This prospective population-based study estimates incident HT and its determinants. With the increasing burden of CVDs and with HT being a major independent risk factor, health planners and policymakers in India must estimate the incident cases of HT and identify those who are at a higher risk of developing HT in order to target preventive strategies and thus meet public health objectives of reducing the burden of HT and CVDs.

The present study found that during the 8-year period from 2007 to 2015, the incidence of HT in individuals aged 45 years and older was 20.8%, with one in five individuals having developed incident HT during this period. Pre-hypertensive individuals had an overall incidence rate of 31.1 per 1,000 (95% CI: 26.20–35.9), and their risk of developing incident HT was 2.24 odds times higher (95% CI: 1.65–3.03) compared to those who were normotensive. Women who were 45–74 years old, educated up to primary level, overweight/obese, and current tobacco users were more likely to develop HT than their respective counterparts. Similar to other studies, our findings also confirm that age was associated with a higher risk of incident HT among women. Those over 45 years had a significantly higher incidence of HT when compared to those below the age of 45 years among women; however, age was not found to be significantly associated with the incidence of HT in the case of men. Our findings are in line with the earlier findings documented in the literature (20–23).

Very few studies in India have investigated the incidence of HT in the general population, with the reported incidence ranging from 12–34 per 1,000 people (24). A population-based study based on three large cities in South Asia estimated an HT incidence of 9.6 per 100 people in Delhi, 7.6 per 100 people in Chennai, and 7.0 per 100 people in Karachi (25). Studies from countries such as China and South Korea have reported an incidence rate of 53 per 1,000 people (26, 27). A recent population-based cohort study estimated an annual incidence rate of HT of 2.5 per 100 among adults aged 40–65 years in Australia (28). However, caution is advised when comparing results from studies that report incidence rates of HT due to the lack of standardization in terms of age and population considerations, criteria for determining HT, duration of study, etc.

The overall 8-year incidence rate of HT in our study was 20.8% in Indian adults aged 45 years and older. Our findings suggest that pre-hypertensives were at over two times greater risk of developing HT, with a rate of 39 per 1,000 people (95% CI: 32.8–45.0) as compared to normotensive individuals who had a rate of 18 per 1,000 people (95% CI: 13.8–22.3). These results are consistent with several other studies that have reported a greater risk of developing HT among pre-hypertensive individuals (21, 23, 28, 29). However, in contrast to our study, a WHO-SAGE longitudinal study from Ghana reported a reduction in the prevalence of HT (reduction in DBP) over a 12-year period, partly explained by greater awareness of HT among the study participants and increased health insurance coverage over time (30). As observed in our study, a higher incidence of HT among pre-hypertensives may be explained by the higher presence of factors such as higher education, urban residence, obesity, diabetes, use of tobacco, alcohol consumption, and higher wealth quintile status among them as compared to those with normal HT. High incident rates of HT among pre-hypertensives, coupled with aging, increasing obesity, and high prevalence of diabetes, are a major cause for concern for policymakers as this cocktail is likely to increase the prevalence of HT and CVD mortality and morbidity in India.

To achieve the target of a 25% relative reduction in HT by 2025, the Government of India has launched the India Hypertension Control Initiative (IHCI). The guidelines on HT screening in India include both opportunistic screening and targeted screening. Opportunistic screening is recommended for those over 18 years of age at every contact with the health system, including with community health workers. On the other hand, targeted screening for HT is aimed at detecting HT among high-risk populations at the community level (31).

The results obtained from our study can be extremely useful in guiding effective interventions among pre-hypertensives who are at higher risk of developing HT. There is sufficient evidence that screening for HT does provide health benefits, especially among high-risk adults (32, 33). In addition to routine and opportunistic screening, early prevention of HT is possible by identification of pre-hypertensives, through strategies such as targeted screening, thus reducing the risk of developing HT. Similarly, prompt treatment of pre-hypertensives can be initiated for those who develop HT. The implications of our findings for both primary and secondary levels of prevention are obvious and are likely to minimize complications and adverse outcomes associated with untreated HT. In addition, the importance of monitoring particularly those with pre-HT cannot be overemphasized. As there are no current guidelines specifically on pre-HT in India (34), it is recommended that guidelines specific to pre-HT are developed and acted upon, with necessary training imparted to the front-line staff.

This approach aims to mitigate the risk of high BP and, subsequently, the risk of CVDs in this population as there is evidence to suggest that controlling BP can avert one-third of CVD mortality (34). Unfortunately, given the huge income inequalities, high out-of-pocket payments, weak government health services, unaffordability of private-sector health services, and lack of pre-HT screening guidelines, it is inevitable that a significant proportion of pre-hypertensives and those at risk of incident HT will remain undiagnosed and will be missed out by the health system. Even among those identified as hypertensives, there are concerns regarding initiation of treatment and compliance to treatment, given the issues around affordability of anti-hypertensive drugs as they are to be purchased through out-of-pocket payments from private pharmacies.

Studies have shown that the treatment rate of HT in India is only 44.0% (35). In the absence of effective monitoring and follow-up, even those who have initiated treatment are less likely to have their blood pressure under control. Again, studies have shown that the control rate of HT in India is 10.4% (35). With high out-of-pocket payments and the predicted rise in incident HT, the economic burden of HT and CVDs on households and its impact on the health systems could be significant.

It may also be noted that the association of risk factors of HT has commonly been reported in prevalence studies but not from incident data. Unlike other cross-sectional studies which provide estimates for a single point in time, the strength of our study is that it uses longitudinal data and reports incident data and risk factors from a prospective perspective. This study therefore contributes to the empirical knowledge of incident HT literature from an LMIC context. Although this is a strength of our study, there are also certain limitations to it. These include the small sample size, restricting the generalizability of the study findings, and the long follow-up of over 8 years, resulting in loss to follow-up. Although the BP measurement was taken multiple times, this was undertaken on a single visit, which may affect the reliability of the research findings by overestimating the incidence of HT (36). In addition, information on comorbidities such as stroke, diabetes, and chronic lung disease, treatment seeking, etc., is self-reported and may have been open to recall bias since memory limitations of the participants may have led to underreported health conditions and may have skewed medical history and lifestyle factors, affecting research reliability. In addition, we did not attempt to differentiate recall bias, if any, between comparison groups. Lastly, the study did not include biometric measures such as triglycerides, cholesterol, and uric acid to assess their impact on the incidence of HT.

## Conclusion

This prospective population-based study conducted in India reveals a worrisome incidence rate of HT among individuals aged 45 and older, with one in five people having developed HT over an 8-year period. Pre-hypertensive individuals face more than twice the risk of HT compared to normotensive individuals, while factors such as lower educational levels, overweight/obesity, and older age exacerbate this risk. Women are particularly vulnerable. The study emphasizes the urgent need for targeted preventive strategies, including opportunistic screening of pre-hypertensives and other high-risk individuals. Although the guidelines on HT screening in India do include both opportunistic screening and targeted screening, challenges within the healthcare system in India, such as income disparities and limited access to affordable treatment, pose significant obstacles to effective HT management. It is recommended that effective implementation of screening programs, both at the point of health service utilization and at the community level, to detect pre-HT and initiate necessary treatment, would be a cost-effective approach to address the growing burden of HT and associated CVDs in India.

## Data Availability

Publicly available datasets were analyzed in this study. These data can be found here: https://www.iipsindia.ac.in/content/Lasi-wave-i.
