# Supplementary material for: A longitudinal study of incident hypertension and its determinants in Indian adults aged 45 years and older: evidence from nationally representative WHO-SAGE study (2007–2015)
Source: Front Cardiovasc Med. 2023 Nov 14;10:1265371. doi: 10.3389/fcvm.2023.1265371 (PMC10682706; doi:10.3389/fcvm.2023.1265371)
Supplement: Supplementary file 1 [file Table1.docx]

**Supplementary Table**

| Table S1. Differences in socio-demographic characteristics of included and excluded sample in the study | | | | |
| --- | --- | --- | --- | --- |
| **Baseline background characteristics** | Excluded sample (n=447) | Included sample (n=3,183) | Difference | p-value |
| **Individual factors** | % | % |  |  |
| **Age groups** |  |  |  |  |
| <45 Years | 14.8 | 16.4 | -1.6 | p=0.726 |
| 45-54 | 31.1 | 31.2 | -0.1 |  |
| 55-64 | 36.7 | 34.0 | 2.7 |  |
| 65-74 | 15.7 | 16.0 | -0.3 |  |
| 75+ | 1.8 | 2.4 | -0.6 |  |
| **Sex** |  |  |  |  |
| Men | 45.9 | 47.5 | -1.6 | p=0.507 |
| Women | 54.1 | 52.5 | 1.6 |  |
| **Education** |  |  |  |  |
| No education | 48.9 | 47.4 | 1.5 | p=0.484 |
| Primary | 22.6 | 26.8 | -4.2 |  |
| Secondary | 11.3 | 11.2 | 0.1 |  |
| Higher | 17.2 | 14.5 | 2.7 |  |
| **Working status** |  |  |  |  |
| Never worked | 32.1 | 26.0 | 6.1 | p=0.117 |
| Not currently working | 21.7 | 22.1 | -0.4 |  |
| Currently working | 46.2 | 51.9 | -5.7 |  |
| **Marital status** |  |  |  |  |
| Currently married | 82.8 | 80.7 | 2.1 | p=0.305 |
| Others | 17.2 | 19.3 | -2.1 |  |
| **BMI categories** |  |  |  |  |
| Normal (18.5-24.9 kg/m^2^) | 51.1 | 52.8 | -1.7 | p=0.834 |
| Underweight (<=18.4) | 33.0 | 32.9 | 0.1 |  |
| Overweight/obese (>=25) | 15.9 | 14.4 | 1.5 |  |
| **Stroke** |  |  |  |  |
| No | 2.7 | 2.8 | -0.1 | P=0.935 |
| Yes | 97.3 | 97.2 | 0.1 |  |
| **Diabetes** |  |  |  |  |
| No | 9.8 | 9.8 | 0 | P=0.839 |
| Yes | 90.2 | 90.2 | 0 |  |
| **Chronic lung disease** |  |  |  |  |
| No | 3.1 | 2.6 | 0.5 | P=0.431 |
| Yes | 96.9 | 97.4 | -0.5 |  |
| **Lifestyle factors** |  |  |  |  |
| **Moderate activity** |  |  |  |  |
| No | 74.2 | 65.6 | 8.6 | p=0.009 |
| Yes | 25.8 | 34.4 | -8.6 |  |
| **Vigorous activity** |  |  |  |  |
| No | 80.5 | 74.7 | 5.8 | p=0.051 |
| Yes | 19.5 | 25.3 | -5.8 |  |
| **Tobacco use** |  |  |  |  |
| Never | 49.8 | 49.1 | 0.7 | p=0.908 |
| Former | 2.7 | 3.6 | -0.9 |  |
| Occasional | 2.7 | 2.5 | 0.2 |  |
| Current | 44.8 | 44.7 | 0.1 |  |
| **Alcohol use** |  |  |  |  |
| No | 85.5 | 84.1 | 1.4 | p=0.568 |
| Yes | 14.5 | 15.9 | -1.4 |  |
| **Household factors** |  |  |  |  |
| **Wealth quintiles** |  |  |  |  |
| Poorest | 18.1 | 22.7 | -4.6 | p=0.405 |
| Poorer | 22.2 | 22.3 | -0.1 |  |
| Middle | 22.2 | 19.4 | 2.8 |  |
| Richer | 18.1 | 19.1 | -1.0 |  |
| Richest | 19.5 | 16.5 | 3.0 |  |
| **Caste** |  |  |  |  |
| SC/ST | 9.8 | 25.5 | -15.7 | p<0.001 |
| OBC | 26.4 | 59.3 | -32.9 |  |
| Others | 63.8 | 15.2 | 48.6 |  |
| **Religion** |  |  |  |  |
| Hinduism | 40.3 | 84.2 | -43.9 | p<0.001 |
| Islam | 7.4 | 11.9 | -4.5 |  |
| Others | 52.3 | 3.9 | 48.4 |  |
| **Place of residence** |  |  |  |  |
| Urban | 30.2 | 20.5 | 9.7 | p<0.001 |
| Rural | 69.8 | 79.5 | -9.7 |  |
|  |  |  |  |  |
| **Total** | **100** | **100** |  |  |
